# Coexistence of Hypertrophic Cardiomyopathy and Arterial Hypertension: Current Insights and Future Directions

**DOI:** 10.3390/diseases14010001

**Published:** 2025-12-22

**Authors:** Vasiliki Katsi, Konstantia Papadomarkaki, Konstantinos Manousiadis, Epameinondas Triantafyllou, Christos Fragoulis, Konstantinos Tsioufis

**Affiliations:** 1 First Cardiology Department, Hippokration General Hospital of Athens, 11527 Athens, Greece; vkkatsi@yahoo.gr (V.K.); christosfragoulis@yahoo.com (C.F.);; 2 Emergency Department, Hippokration General Hospital of Athens, 11527 Athens, Greece; kmanous@med.uoa.gr; 3 Department of Cardiology, Hippokration General Hospital of Athens, 11527 Athens, Greece

**Keywords:** hypertrophic cardiomyopathy, hypertension, comorbidities, treatment strategy

## Abstract

Background: Hypertrophic cardiomyopathy (HCM) is the most common inherited cardiac disease. Arterial hypertension represents the leading modifiable risk factor for cardiovascular morbidity and mortality globally. Their coexistence is frequent, affecting approximately 40–60% of adults with HCM, yet the implications of this overlap remain insufficiently investigated. Methods: We conducted a narrative review of the existing literature addressing the clinical profile and management strategies in patients with concomitant HCM and hypertension. Particular emphasis was placed on pharmacologic treatment and the role of emerging therapies for this population. Results: Patients with both conditions are generally older, with more cardiometabolic comorbidities and greater functional limitation than those with isolated HCM. Hypertension may confound diagnosis and is linked to a higher prevalence of atrial fibrillation and stroke. Its effect on ventricular arrhythmias, sudden cardiac death and mortality is less clear. Management is challenging, as vasodilatory antihypertensives can exacerbate left ventricular outflow tract obstruction. β-blockers and non-dihydropyridine calcium channel blockers are preferred, while novel agents such as myosin inhibitors and SGLT2 inhibitors show potential but require further study. Conclusions: The coexistence of HCM and hypertension is frequent but insufficiently studied, with major implications for diagnosis and treatment. Further research is essential to optimize management and outcomes.

## 1. Introduction

Hypertrophic cardiomyopathy (HCM) is the most common form of inherited cardiac disease with an estimated prevalence of approximately 1:500 in the general population [1,2]. According to the recent guidelines, it is defined by left ventricular (LV) hypertrophy not explained by increased loading conditions, with a maximal wall thickness >15 mm in any myocardial segment, while in first-degree relatives, a threshold of >13 mm is considered diagnostic [1,2]. HCM has a strong genetic component, with pathogenic variants in sarcomeric genes identified in a significant proportion of patients. Around 30–40% of cases are attributable to such variants, most commonly inherited in an autosomal dominant manner with incomplete penetrance and variable expressivity.

Over the past four decades, a steady rise in HCM diagnoses worldwide has reshaped perceptions of the disease and its epidemiology. Once considered a rare disease of the young with an ominous prognosis, HCM is now viewed as a manageable chronic condition, often compatible with normal life expectancy. This evolution in the disease profile is attributed to improved diagnostic capabilities, greater public awareness, and advances in HCM management. The mean age at diagnosis has increased from 36 years before 1982 to 44 years after 1992, with more recent data (SHaRe registry) indicating a mean diagnostic age of 51 ± 16 years [3]. Patients are now older at presentation, often asymptomatic, and tend to exhibit milder phenotypes with more frequent negative or inconclusive genetic testing. HCM is increasingly diagnosed in middle-aged and older adults, burdened by several cardiovascular comorbidities that may influence treatment decisions and clinical outcomes.

Hypertension is the most important modifiable risk factor for all-cause and cardiovascular morbidity and mortality, affecting over one billion people worldwide [4]. Hypertension coexists in approximately 40–60% of adults with HCM, an overlap that poses both diagnostic challenges and therapeutic dilemmas. In hypertensive patients with milder disease phenotypes, left ventricular hypertrophy (LVH) may initially be attributed to hypertension rather than underlying HCM. At the same time, it has been proposed that antihypertensive therapy may mitigate or postpone the clinical expression of the HCM phenotype [5].

Management is particularly complex since first-line antihypertensive agents, such as angiotensin-converting enzyme (ACE) inhibitors, angiotensin receptor blockers (ARBs) and dihydropyridine calcium channel blockers (CCBs), possess vasodilatory effects that can worsen left ventricular outflow tract (LVOT) obstruction, present in up to 70% of HCM patients and exacerbate symptoms. Despite this frequent overlap, the clinical course and outcomes of patients with both conditions remain insufficiently investigated and robust evidence guiding the co-management of HCM and hypertension is lacking, constraining clinical decision-making and optimization of outcomes [6]. We sought to review the existing literature on the clinical profile and disease course of adults with co-occurring HCM and hypertension, with a particular focus on pharmacologic management in the context of emerging therapies for hypertrophic cardiomyopathy. The knowledge and evidence gaps highlighted can inform future research and contribute to refining clinical management strategies to achieve optimal outcomes in this patient population.

## 2. Clinical Characteristics and Prognosis

### 2.1. Demographics and Clinical Characteristics

Across observational cohorts, HCM patients with co-occurring hypertension (HT) are consistently older than those with isolated HCM, have a lower prevalence of family history of HCM and sudden cardiac death (SCD) and carry a higher burden of cardiometabolic disease, including diabetes, obesity, hyperlipidemia and coronary artery disease (Table 1) [6,7]. Prevalence by sex is generally similar overall, although age-stratified differences have been described: in a cohort with a median age of 55, HT was more frequent in men ≤37 years and women ≥75 years [8]. In the limited studies that recorded race, co-existing HT is reported more frequently among Black adults with HCM. Sheikh et al [9] reported a higher prevalence of hypertension among Black adults with HCM, a group that is also disproportionately affected by diagnostic disparities and delays in access to specialized care, compared with their White counterparts. However, evidence regarding racial differences in hypertension prevalence within HCM populations remains inconclusive, in part because Black adults continue to be underrepresented in both clinical care settings and research cohorts.

### 2.2. Functional Status and Symptoms

In terms of symptom burden, manifestations of congestion such as paroxysmal nocturnal dyspnea and ankle edema are reported more frequently with HT, while chest pain is a more prominent complaint in this group [6,7]. Functional capacity appears to be more frequently compromised in the presence of hypertension in patients with HC. In comparative analyses, New York Heart Association (NYHA) functional class has overall been reported as more advanced in the hypertensive subgroup, with some studies identifying hypertension itself as an independent predictor of higher NYHA class [7]. Exercise performance, assessed by metabolic equivalents (METs) and peak oxygen consumption (VO_2_), tends to be lower in hypertensive HCM cohorts.

HCM patients with hypertension tend to experience lower rates of pre-syncope and syncope, an observation consistent across several datasets. This may be partly attributed to the higher baseline blood pressure in these patients, which could provide a greater “reserve” for maintaining consciousness during syncope events. The increased baseline blood pressure may help counterbalance the drop in blood pressure caused by abnormal reflex regulation of the blood vessels, thus lowering the likelihood of syncope [9].

### 2.3. Disease Models for Studying Hypertrophic Cardiomyopathy and Arterial Hypertension

Recent advancements in disease modeling have greatly deepened our understanding of hypertrophic cardiomyopathy and arterial hypertension. Using a variety of human-derived and animal models, alongside computational simulations, researchers have been able to dissect the underlying mechanisms more precisely. For instance, human induced pluripotent stem cell (iPSC)-derived cardiomyocytes provide a unique window into patient-specific genetic and molecular nuances of HCM [10]. Transgenic rodents bearing sarcomeric mutations mirror many aspects of HCM, allowing us to track disease progression and test treatments. Hypertensive cardiac remodeling has been effectively recapitulated using pressure- overload models such as transverse aortic constriction and DOCA-salt, giving valuable insights into hypertensive heart disease [9]. When these genetic and hypertensive stressors are combined in models, the phenotypic overlaps become clearer, aiding the study of their interplay [11]. Computational models augment these approaches by simulating ventricular remodeling and wall stress, which help predict disease trajectories. Ex vivo myocardial tissue analyses provide rich structural and functional data, bridging the lab bench to clinical reality. Together, these models offer powerful tools that enrich our mechanistic insights and support the development of novel therapeutic strategies targeting the complex relationship between HCM and hypertension.

### 2.4. Atrial Fibrillation and Stroke

Atrial fibrillation (AF) is the most frequent sustained arrhythmia in HCM and constitutes a major clinical complication with significant impact on the disease course. Lifetime prevalence estimates suggest that 20–25% of HCM patients will develop AF, with an annual incidence of 2–4%, a rate nearly six-fold higher than in the general population of similar age [12]. AF is associated with impaired quality of life, increased morbidity, as well as increased cardiovascular and all-cause mortality, largely driven by a higher incidence of heart failure progression and thromboembolic stroke.

HCM patients with concurrent hypertension exhibit a higher burden of AF compared to their normotensive counterparts [7,13,14]. Prevalent AF and a prior history of stroke or transient ischemic attack are more frequently documented in the hypertensive subgroup. Notably, the presence of prevalent AF at baseline has not been shown to independently predict stroke development in patients with HCM [13]. This observation underscores that additional mechanisms may contribute to the elevated thromboembolic risk in this population. Beyond hypertrophy itself, structural abnormalities such as apical aneurysms, myocardial crypts, and areas of non-compaction may serve as niduses for thrombus formation and embolic events [13].

A validated risk prediction model, the HCM-AF score, was recently introduced for estimating the risk of developing AF in patients with HCM. Τhis tool, developed in a large and diverse cohort at the Tufts HCM Center, uses four readily available clinical variables—transverse left atrial dimension, age at current evaluation, age at initial HCM diagnosis, and presence of heart failure symptoms—to provide individualized 2- and 5-year AF risk estimates. Although this score does not take into account the presence of hypertension (unlike other AF risk scores used in the general population), available data suggest that hypertension represents a significant risk marker for AF in HCM patients that warrants closer surveillance [15]. The identification of such associations between common cardiovascular risk factors, including hypertension, underscores the importance of addressing their management with equal diligence as condition-specific complications.

Notably, in many studies evaluating the association between hypertension and the incidence of stroke in patients with HCM, the etiology of stroke is not specified. Therefore, the relative contributions of AF-related embolic events versus hypertension-related (atherothrombotic or hemorrhagic) cerebrovascular disease remain incompletely understood [6].

### 2.5. Ventricular Arrhythmias and Sudden Cardiac Death

HCM is a condition that intrinsically carries a well-recognized predisposition to ventricular arrhythmias and SCD; however, the evidence on whether systemic hypertension modifies either risk remains inconclusive. Some series report a greater frequency of non-sustained ventricular tachycardia among hypertensive HCM patients [5], whereas in others, a history of resuscitated ventricular fibrillation/arrest and implantable cardioverter-defibrillator [ICD] prevalence are observed more often in non-hypertensive HCM cohorts [7].

The association of hypertension and elevated systolic blood pressure (SBP) with increased risk of SCD is well-supported by robust epidemiological data. According to a recent meta-analysis, individuals with hypertension exhibit a more than twofold higher risk of SCD (RR = 2.10, 95% CI: 1.71–2.58), while each 20 mmHg increment in SBP is linked to a 28% increase in SCD risk (RR = 1.28, 95% CI: 1.19–1.38) [16]. Nevertheless, the coexistence of these two conditions does not appear to confer an additive or synergistic increase in SCD risk. When adjusted for age, comorbidities, and established HCM-specific risk factors, hypertension does not emerge as an independent predictor of SCD in this population.

### 2.6. Cardiovascular Death and All-Cause Mortality

A notable finding is that, although adults with both HCM and hypertension have a less favorable clinical profile—being older and having more cardiovascular comorbidities—overall outcomes, including all-cause mortality and cardiovascular events, are broadly similar to those with HCM alone. Most adjusted analyses show no significant associations between hypertension and clinical outcomes [6,7]. A possible explanation for this discrepancy is that access to specialized HCM care leads to more individualized management and a timely response to clinical changes. Another is that hypertension in the context of HCM may involve distinct mechanisms, such as increased myocardial contractility rather than elevated peripheral resistance [6].

**Table 1:** Comparison of the clinical characteristics, risk factors, and outcomes of patients with hypertrophic cardiomyopathy (HCM) alone versus those with concurrent HCM and arterial hypertension. BP = blood pressure; CAD = coronary artery disease; CMR = cardiac magnetic resonance; LGE = late gadolinium enhancement; LVH = left ventricular hypertrophy; NYHA class = New York Heart Association class; SCD = sudden cardiac death.

Aspect	HCM Alone	HCM with Hypertension	Clinical Implications	Key References
Average Age	Younger (mean around 36–51 years)	Older (greater age at diagnosis)	Older age increases comorbidities and affects treatment tolerance	Canepa 2020 [3]; Arabadjian 2024 [6]
Cardiometabolic Comorbidities	Lower prevalence	Higher prevalence (diabetes, obesity, CAD)	Requires comprehensive management of additional risks	Lopes 2023 [7]; Arabadjian 2024 [6]
Functional Status	Better functional capacity	More advanced NYHA class, worse exercise capacity	Higher symptom burden	Arabadjian 2024 [6]
Atrial Fibrillation	20–25% lifetime prevalence	Increased burden	Greater stroke risk and need for arrhythmia surveillance	Arabadjian 2024 [6]; Zörner 2024 [14]
Syncope/Presyncope	More frequent	Less frequent	Hypertension may provide BP reserve during syncope	Arabadjian 2024 [6]
Ventricular Arrhythmias & SCD	Well-established risk	Conflicting evidence on additional risk	Risk stratification remains complex	Wang 2023 (PeerJ) [10]; Pan 2020 [17]
Mortality and Cardiovascular Events	Baseline risk	Similar after adjustment	Specialized care optimizes outcomes	Arabadjian 2024 [6]; Lopes 2023 [7]
Imaging Features	More likely asymmetric LVH	Mixed patterns, concentric LVH also common	Highlights diagnostic complexity; importance of CMR/LGE and LGE	Tarkiainen 2025 [18]; Rodrigues 2016 [19]; Yang 2024 [20]

## 3. Hypertension as a Modifier of Disease Expression in Hypertrophic Cardiomyopathy

Hypertension imposes chronic pressure overload that markedly influences HCM progression by impairing myocardial energy efficiency and increasing oxygen demand. Sustained high afterload drives maladaptive remodeling, characterized by elevated oxygen consumption and reduced contractile efficiency [17]. Moreover, hypertension alters sarcomeric protein expression, potentially intensifying genotype-phenotype associations in HCM and accelerating disease progression through heightened mechanical stress on cardiomyocytes [21]. The pattern of fibrosis differs between the two conditions: hypertensive hearts generally exhibit diffuse interstitial and replacement fibrosis, while sarcomeric HCM is more typified by patchy fibrosis associated with myocyte disarray [22]. Microvascular dysfunction further complicates this interplay by impairing diastolic coronary perfusion, thereby worsening ischemia and promoting fibrotic remodeling [23]. Collectively, these mechanisms demonstrate how hypertension can serve as a significant modifier of HCM’s clinical course.

## 4. Cardiovascular Magnetic Resonance

Cardiovascular magnetic resonance (CMR) represents an integral component of the comprehensive evaluation of cardiac disease, increasingly employed to establish diagnosis, elucidate the underlying etiology, and provide prognostic insights. CMR is regarded as the gold standard imaging modality for the evaluation of cardiac morphology and function. It offers reproducible and highly accurate assessment of myocardial wall thickness, mass, chamber volumes and global systolic performance, allowing at the same time non-invasive fine tissue characterization.

The diagnosis of HCM is based on the presence of unexplained LVH. Since hypertension is the most common cause of LVH, its presence can pose diagnostic challenges in the case of mild or atypical phenotypes of HCM. On morphological analysis, images obtained with steady-state free precession imaging cine sequences allow precise delineation of the extent and pattern of hypertrophy.

The basal anterior septum, in continuity with the anterior free wall, represents the most common location for hypertrophy in HCM; nevertheless, nearly any pattern and distribution of LV wall thickening can be observed. In some cases, hypertrophy can be focal, confined to only 1 or 2 LV segments, occasionally with preserved LV mass [2]. CMR is particularly useful for identifying such localized forms, especially in regions less accessible to echocardiography, including the anterolateral wall, posterior septum, and apex. It can also detect additional phenotypic features such as myocardial crypts, apical aneurysms, hypertrophied or apically displaced papillary muscles, elongation of the anterior mitral leaflet, anomalous papillary muscle insertion without chordae tendineae, myocardial bridging, and right ventricular hypertrophy, findings that are suggestive of HCM.

Although classic imaging features may point to a specific diagnosis, these are not always sensitive or specific. The pattern of hypertrophy in HCM is heterogeneous and not invariably asymmetric. Concentric LVH—traditionally considered the hallmark of hypertensive heart disease (HHD—may also occur in HCM patients [6,24]. Conversely, asymmetric hypertrophy in the setting of HHD occurs more frequently than previously recognized, with asymmetric LVH of the basal or mid-septum reported in up to 21% of a hypertensive cohort [18].

Beyond the different hypertrophy patterns, additional CMR-derived parameters have been explored mainly in the differential diagnosis between HCM and HHD. These include the extent of diffuse fibrosis assessed by native T1 mapping and myocardial extracellular volume (ECV); myocardial deformation indices such as global radial strain (GRS), global circumferential strain (GCS), and global longitudinal strain (GLS); and markers of hypertrophy and systolic–diastolic abnormalities, including left ventricular mass index (LVMI), maximal left ventricular wall thickness (LVWT), and end-diastolic and end-systolic volume index (EDVI, ESVI). Few studies, though, have examined the impact of the coexistence of the two conditions on these parameters.

In a recent study, a stepwise reduction in LV GRS and GLS was observed across controls, HCM patients alone, and HCM patients with HT. HT was negatively associated with GCS and LVEF, with a higher proportion of HCM patients with LVEF <55% in those with HT compared to those without, although mean LVEF was similar between the groups [19].

The principal value of CMR lies in its ability to provide non-invasive tissue characterization. This is essential not only for differentiating HCM from other hypertrophic phenocopies, such as cardiac amyloidosis or Anderson–Fabry disease, but also for identifying markers of poor prognosis, including progression to the ‘dilated phase’ of HCM or the development of malignant ventricular arrhythmias.

### 4.1. Late Gadolinium Enhancement

The presence and distribution of late gadolinium enhancement (LGE) provide important diagnostic and prognostic insights in HCM. The most commonly observed pattern is focal enhancement at the right ventricular insertion points (anterior, inferior, or both), often accompanied by diffuse, patchy, or hazy mid-wall enhancement within hypertrophied segments, but it can be transmural in case of apical aneurysms. LGE is a common finding in sarcomeric HCM, its prevalence ranging between 40% and 95% depending on the average age of the studied population.

The quantitative extent of LGE has a prognostic role, as it is independently associated with an increased risk of SCD and adverse outcomes, including progression to heart failure [20]. Notably, an LGE ≥15% of left ventricular (LV) mass confers more than a twofold increase in SCD risk, even among patients otherwise classified as low-risk based on conventional clinical criteria [25].

Although it is not incorporated in the HCM Risk SCD score, proposed by the ESC guidelines to evaluate the 5-year risk of sudden cardiac death, current guidelines recommend consideration of LGE burden in risk stratification and decision-making regarding implantable cardioverter-defibrillator (ICD) therapy.

In LVH of hypertensive etiology, LGE lesions are present in up to 50%, in a non-specific, non-subendocardial pattern, lacking a characteristic coronary vascular distribution, and are often less extensive than in HCM [26,27]. Of note, the presence of hypertension in the setting of HCM does not seem to affect the phenotype of LVH or the pattern of LGE lesions [19].

Current international guidelines from both the European Society of Cardiology (ESC) and the American College of Cardiology (ACC) assign CMR a Class I recommendation in the evaluation of HCM, particularly when echocardiography yields inconclusive findings, when uncertainty about the hypertrophic phenotype persists, or when additional clarification is required [1,2].

### 4.2. Imaging Markers to Distinguish HCM from Hypertensive LVH

Distinguishing HCM from hypertensive LVH can be challenging, especially in older adults with concentric hypertrophy. Multimodality imaging, including echocardiography and CMR, helps improve diagnostic accuracy. Several features favor HCM: asymmetric hypertrophy (particularly basal anterior septum), presence of myocardial crypts, apical aneurysm or apical hypertrophy, elongated anterior mitral leaflet, anomalous papillary muscle insertion, and focal patchy LGE. Conversely, hypertensive heart disease more commonly shows concentric LV thickening and diffuse interstitial fibrosis. Quantitative CMR markers such as native T1 and extracellular volume fraction (ECV) have been shown to discriminate HCM from hypertensive LVH at a group level—with higher native T1/ECV in genotype-positive HCM and in regions of replacement fibrosis [28]. Likewise, LGE distribution (patchy, often mid-wall or at RV insertion points) and extent better characterize sarcomeric disease [29]. Myocardial strain measured by speckle-tracking echocardiography or CMR feature tracking also provides additive diagnostic value: HCM typically demonstrates more pronounced regional strain abnormalities in hypertrophied segments even when ejection fraction is preserved [30]. Despite these tools, overlap remains and no single parameter is definitive; therefore, integrating clinical history, family history, ECG features, genetic testing, and multimodality imaging provides the highest diagnostic yield.

### 4.3. Myocardial Ischemia in HCM and Hypertensive Left Ventricular Hypertrophy

Microvascular remodeling and vessel rarefaction differ fundamentally between HCM and hypertensive LVH, influencing myocardial perfusion and ischemic risk. Hypertension causes structural remodeling that thickens vessel walls and reduces vessel density, increasing vascular resistance and reducing coronary flow reserve [31]. In HCM, microvascular remodeling occurs alongside extramural coronary compression due to myocardial bridging and septal hypertrophy, exacerbating supply–demand mismatch [32]. Stress perfusion CMR detects heterogeneous subendocardial ischemia in HCM attributable to dynamic obstruction and increased metabolic demands [33], whereas hypertension tends to produce more diffuse perfusion defects reflective of global microvascular dysfunction [31]. Prognostically, ischemia in HCM correlates strongly with heart failure progression and sudden cardiac death risk [33,34], while in hypertensive LVH, ischemia predicts heart failure worsening and cardiovascular events but is less linked to arrhythmic risk. PET and invasive studies in HCM have linked coronary microvascular dysfunction with long-term remodeling and progression to systolic dysfunction [35], while transthoracic Doppler coronary flow velocity reserve (CFVR) provides independent prognostic information [36]. Appreciating these mechanistic and imaging distinctions is essential for personalized patient management.

## 5. Medical Management

In patients with symptomatic HCM, pharmacological treatment is primarily aimed at symptom relief and functional improvement, although there is limited evidence that medical therapy alters the long-term natural history of the disease or significantly reduces the risk of SCD [37].

While several therapeutic options exist for HCM, the focus of this review is limited to those with potential implications for concomitant hypertension (Table 2). Beta-blockers, CCBs and diuretics are of particular interest, given their dual role as both HCM therapies and antihypertensive agents. Similarly, emerging treatments such as mavacamten, which is being studied in hypertensive populations, and SGLT2 inhibitors, which are under investigation for potential benefit in non-obstructive HCM and possess mild antihypertensive properties, warrant discussion in this context. Disopyramide and ranolazine, as well as invasive septal reduction strategies, have an established role in HCM management but no clear relevance to blood pressure control; therefore, they are not addressed here.

### 5.1. Beta-Blockers

Both European and American guidelines recommend non-vasodilating β-blockers—such as metoprolol, bisoprolol, and nadolol—as first-line pharmacologic agents in patients with obstructive HCM. β-blockers also have a well-established role in the treatment of hypertension, and although they are not considered first-line antihypertensive therapy, their administration in patients with concomitant HCM and hypertension offers the advantage of addressing both conditions simultaneously. Dynamic LVOT obstruction is a complex pathophysiological hallmark of HCM, defined by a gradient exceeding ≥50 mm Hg at rest or during exercise, that occurs in approximately 70% of patients with HCM. In the majority of these cases, the outflow obstruction is caused by systolic anterior movement (SAM) of anomalous mitral valve leaflets, contacting the septum at the subaortic level. Less frequently, obstruction may occur in the absence of SAM, as a result of midcavity muscular apposition, usually caused by anomalous papillary muscle insertion directly into the anterior mitral leaflet without interposed chordae. By reducing heart rate, β-blockers prolong diastole, thereby improving ventricular filling, enhancing coronary perfusion, and lowering myocardial oxygen demand—all particularly beneficial in the context of hypertrophied and stiff myocardium. The reduction in myocardial contractility is believed to decrease the inotropic state of the left ventricle and alleviate SAM of the mitral valve, ultimately reducing the LVOT gradient. β-blockers can alleviate symptoms such as angina and dyspnoea, often resulting from microvascular ischaemia, impaired diastolic function, and elevated ventricular filling pressures and are therefore recommended for symptomatic patients, irrespective of the presence or severity of intraventricular obstruction [38].

Beyond their hemodynamic effects, β-blockers also provide anti-arrhythmic benefit by attenuating sympathetic drive, thus reducing the incidence of both supraventricular and ventricular arrhythmias [39].

β-blockers have historically served as the first-line treatment for dynamic LVOT obstruction in HCM (HOCM), based on observational data and clinical experience. It was only recently that the first randomized controlled trial assessing β-blockers in this context was conducted. Compared with placebo, metoprolol significantly reduced resting, exercise-induced and post- exercise-induced LVOT gradient and improved NYHA functional class, quality of life and global longitudinal strain of the left ventricle. No significant changes in exercise capacity, peak oxygen consumption (VO_2_ peak), or N-terminal pro-B-type natriuretic peptide (NT-proBNP) levels were noticed [40,41].

Nevertheless, results from the recently published MAPLE-HCM trial challenge the traditional reliance on β-blockers in obstructive HCM. In this randomized, double-blind, phase III study comparing monotherapy with cardiac myosin inhibitor aficamten versus metoprolol in symptomatic oHCM, treatment with metoprolol showed no benefit in exercise capacity as evidenced by the decline in pVO_2_ over 24 weeks (mean change −1.2 mL/kg/min for metoprolol arm) [42].

Current HCM guidelines recommend β-blockers as first-line therapy for symptomatic HCM, both in obstructive and non-obstructive forms. However, the supporting evidence for their use in nHCM is particularly limited, as these recommendations are largely extrapolated from obstructive HCM data and rely more on expert consensus than on robust evidence from well-designed studies. Moreover, emerging data suggest that withdrawal of β-blockers in patients with heart failure with preserved ejection fraction (HFpEF) may improve exercise capacity, at least partly through restoration of chronotropic reserve, given that β-blocker–induced chronotropic incompetence can restrict cardiovascular response during exertion [43]. These observations raise important questions regarding the impact of β-blockers on functional capacity—a fundamental therapeutic goal for symptomatic HCM patients. Collectively, they indicate that the traditional first-line positioning of β-blockers in symptomatic HCM, especially in nHCM, may warrant careful reappraisal.

### 5.2. Calcium Channel Blockers

Non-dihydropyridine CCBs verapamil and diltiazem are recommended as alternatives in patients who are intolerant or have contraindications to beta-blockers. Similar to β-blockers, the primary objective of calcium channel blockade in HCM is symptom relief, achieved by lowering left ventricular (LV) diastolic pressures and enhancing LV filling through heart rate reduction. Increased intracellular calcium concentrations, observed in myocardial tissue of HCM patients undergoing surgery or transplantation, contribute to impaired myocardial relaxation and diastolic dysfunction [44]. Thus, beyond their negative inotropic, chronotropic and dromotropic effect, CCBs may also have a beneficial role in addressing the underlying diastolic abnormalities characteristic of HCM [39].

Non-dihydropyridine CCBs should be used with caution in the presence of hypotension, significant atrioventricular conduction abnormalities, severe outflow tract obstruction, or elevated pulmonary pressures. Notably, for verapamil, it has been shown that its vasodilatory properties may outweigh its negative inotropic action, worsening dynamic LVOT obstruction [45]. In patients with severe dyspnea at rest, hypotension, or very high resting gradients (e.g., >100 mmHg), current guidelines classify verapamil use as potentially harmful, although these recommendations are based on a limited number of studies and case reports (LoE 3C) [2].

Dihydropyridine CCBs are generally avoided in HOCM as a slight decrease in systemic blood pressure may worsen outflow obstruction. LVOT obstruction is dynamic and can be exacerbated by any factors that alter left ventricular loading conditions or myocardial contractility. Variability in the LVOT gradient has been associated with changes in volume status, autonomic nervous activity, pharmacotherapy, exercise, recent cardioplegia, and even patient positioning. Afterload- and preload-reducing drugs—including angiotensin-converting enzyme inhibitors, angiotensin receptor blockers, dihydropyridine CCBs, nitrates, and phosphodiesterase inhibitors—are recommended to be avoided, as they may exacerbate obstruction and worsen symptoms [46]. Although the combined use of CCBs and β-blockers for HCM-specific therapy lacks supporting evidence, this combination may be considered in patients with coexisting hypertension [2].

Of note, low-dose loop or thiazide diuretics can be used cautiously to relieve pulmonary or systemic congestion in HCM patients, with thiazide diuretics also playing a central role in the management of concomitant hypertension; careful titration is essential to avoid hypovolemia and potential consequent worsening of LVOT [1,2].

### 5.3. Cardiac Myosin Inhibitors

Selective cardiac myosin inhibitors (CMIs) have marked the beginning of a new era in the management of HCM, representing the first therapy specifically targeting the underlying pathophysiology of the disease. They are allosteric inhibitors that reduce actin-myosin cross-bridging and counteract an over-activation of cardiac myosin, decreasing contractility and improving myocardial energetics. Mavacamten is the first-in-class agent that gained approval for the treatment of HOCM. It is recommended for patients who remain symptomatic despite optimal first-line therapy with β-blockers or non-dihydropyridine CCBs and may also be considered as monotherapy in case of intolerance or contraindications to standard medical therapy [1,2]. Following the results of the VALOR-HCM trial, mavacamten received regulatory approval as an alternative treatment strategy to delay or avoid septal reduction therapy in patients who meet guideline criteria for invasive intervention.

Elevated systemic blood pressure can minimize the pressure gradient across LVOT, possibly attenuating the clinical benefits of mavacamten in patients with concomitant hypertension. In a post hoc exploratory analysis of the EXPLORER-HCM trial, Wang et al. investigated the efficacy of mavacamten in patients with HOCM and hypertension, a comorbidity present in 47% of trial participants. EXPLORER-HCM was an RCT comparing mavacamten with placebo in 251 patients with HOCM and an elevated LVOT gradient >50 mmHg. Despite differences in baseline clinical profiles between hypertensive and normotensive individuals, the therapeutic benefits of mavacamten regarding improvements in functional capacity, symptoms, quality of life and biomarker profiles were similar across both groups. Furthermore, no interaction was found between systolic blood pressure levels and the treatment effect of mavacamten [47].

Given that the findings stem from a post hoc analysis and that the hypertensive cohort included only individuals with relatively well-controlled blood pressure—who may not be representative of the broader hypertensive population typically encountered in clinical practice—it is evident that the results are hypothesis-generating. Moreover, the limited follow-up duration of 30 weeks further constrains conclusions regarding the long-term effects of myosin inhibition. Nevertheless, currently available data suggest that mavacamten can be safely initiated in patients with grade 1 hypertension, including those on antihypertensive therapy. While mavacamten does not appear to exert direct antihypertensive effects, its ability to reduce LVOT gradients may enable the safer introduction of afterload-reducing agents, which are commonly used in the management of hypertension [48].

Given the growing body of evidence supporting their efficacy, myosin inhibitors are expected to gradually shift current treatment paradigms and may ultimately challenge the traditional position of β-blockers and CCBs as first-line therapies in symptomatic HOCM, while studies in non-obstructive and pediatric populations are still ongoing. The MAPLE-HCM trial mentioned above demonstrated the superiority of aficamten, another cardiac myosin inhibitor, over metoprolol monotherapy in patients with symptomatic HOCM. Although it has not yet received formal regulatory approval, aficamten monotherapy led to significantly greater improvements in peak oxygen uptake, symptom burden, LVOT gradient, NT-proBNP levels, and left atrial volume index compared to metoprolol, which did not significantly improve these objective parameters despite modest gains in subjective symptom scores. Notably, aficamten was associated with a minimal increase in arterial blood pressure, with systolic and diastolic pressures rising by 4.5 mmHg and 2.3 mmHg, respectively, over 24 weeks [42].

The interactions between myosin inhibitors and hypertension are complex, involving mechanistic pathways that remain incompletely understood and represent an important area for future investigation. Their ability to reduce myocardial contractile force and relieve dynamic LVOT obstruction can increase effective stroke volume and potentially unmask or exacerbate hypertension in patients with underlying vascular stiffness. The rise in blood pressure is thought to result from the abrupt improvement in forward flow and afterload-normalized stroke volume once obstruction is relieved.

A recently published case report described accelerated hypertension following mavacamten initiation in a patient with severe obstructive HCM, hypothesizing that rapid relief of LVOT obstruction may alter flow dynamics and increase effective stroke volume, which—particularly in patients with chronic hypertension and increased arterial stiffness—could precipitate accelerated hypertension [49]. Further long-term data and broader clinical experience are needed to better characterize this emerging class of agents and their interactions with common comorbidities in HCM, underscoring the need for vigilant blood pressure monitoring and individualized antihypertensive management during mavacamten initiation.

### 5.4. Sodium-Glucose Cotransporter 2 Inhibitors

Sodium–glucose cotransporter 2 inhibitors (SGLT2i) have recently become an established therapy in heart failure patients, across the full spectrum of ejection fraction and independent of diabetes mellitus status [50,51]. In addition to their glucose-lowering action and mild antihypertensive effects, SGLT2 inhibitors exert cardioprotective effects by enhancing cardiac energetics, optimizing myocardial substrate metabolism, and promoting favorable left ventricular remodeling. Such mechanisms may be especially relevant in HCM, a condition characterized by energetic deficiency and impaired diastolic function, where progression to heart failure remains a predominant cause of morbidity and mortality.

Preclinical evidence supports this concept. In a murine model of HCM with the myosin R403Q mutation, empagliflozin reduced LVH and fibrosis while improving diastolic function and contractile reserve. These benefits were associated with enhanced mitochondrial energetics and substrate metabolism, reduced intracellular sodium and downregulation of maladaptive mTOR signaling, indicating that SGLT2i can directly ameliorate structural and metabolic dysfunction in HCM [11]. Complementary clinical observations further strengthen this rationale. In small open-label studies of diabetic HCM patients, SGLT2i improved symptoms, while a large real-world dataset demonstrated that HCM patients prescribed SGLT2i experienced reduced all-cause mortality, hospitalizations and cardiovascular symptoms, with a favorable safety profile [52]. In the same context, the effect of sotagliflozin, a dual SGLT1/2 inhibitor, on symptoms, functional capacity, and patient-reported outcomes is currently being evaluated in the SONATA-HCM trial [53].

SGLT2 inhibitors emerge as an attractive therapeutic target, with the potential to modify the natural history of HCM and offer early intervention opportunities in genotype-positive individuals before clinical manifestation. In the setting of coexisting hypertension and hypertrophic cardiomyopathy, SGLT2 inhibitors may offer dual benefit through their antihypertensive and metabolic effects; however, their use requires careful consideration given the hemodynamic vulnerabilities of obstructive physiology. SGLT2 inhibitors exert modest natriuretic and osmotic diuretic effects, along with reductions in arterial stiffness and neurohormonal activation [49,50], lowering systemic blood pressure by approximately 3–6 mmHg and improving metabolic milieu through weight loss and improved glycaemia—mechanisms that plausibly influence both LV loading conditions and myocardial energetics [54,55]. An acute reduction in intravascular volume and preload may decrease LV cavity size and, in patients with dynamic LVOT obstruction, transiently augment LVOT gradients and exacerbate symptoms [56]. Conversely, their modest antihypertensive action and beneficial myocardial effects—including reductions in interstitial fibrosis and improvements in myocardial energetics and diastolic function observed in HFpEF and experimental HCM models—may support long-term improvement in diastolic function and remodeling [57,58]. Because formal randomized data in HCM populations are limited, clinicians should individualize SGLT2i use in HCM with hypertension: avoid abrupt volume depletion, start at standard doses, monitor blood pressure and symptoms closely after initiation, and reassess LVOT gradients if new symptoms or hypotension occur. Ongoing clinical trials (including exploratory HCM–specific SGLT2i programs) are needed to define net clinical benefit in this subgroup [59].

Practical Clinical Guidance:

- Start SGLT2i at the recommended dose; counsel patients about orthostatic symptoms [54].
- Monitor blood pressure, weight and symptoms within 1–2 weeks, and recheck LVOT gradient if symptoms worsen [56].
- In patients with labile BP or significant LVOT obstruction, consider initiating therapy under specialist HCM supervision [55].
- Document rationale and reassess LV structure and function if clinically indicated [59].

### 5.5. Angiotensin Receptor Blockers

The role of angiotensin receptor blocker (ARB) therapy in patients with HCM remains under discussion and current evidence is still limited.

Preclinical studies in mouse models of sarcomeric HCM have shown that early treatment with agents such as losartan may attenuate disease progression, provided that it is initiated prior to the development of LVH. Similarly, clinical trials investigating ACE-I and ARBs in human HCM have failed to demonstrate consistent benefits, particularly in older adults with established disease and heterogeneous or undefined genetic backgrounds [60,61,62]. Based on data from the VANISH trial, which demonstrated that early intervention with valsartan led to stabilization or improvement in several parameters of myocardial structure and function [63] the 2024 ACC/AHA guidelines suggest considering valsartan in younger patients (typically ≤45 years old) with nonobstructive HCM, a confirmed pathogenic sarcomeric variant, and a mild phenotypic expression, with the aim of slowing adverse cardiac remodeling. While this approach is not intended for hypertensive control per se, it underscores the potential dual role of certain agents in both blood pressure management and disease trajectory modification.

**Table 2:** Pharmacologic agents discussed regarding their dual implications for HCM and hypertension management. HCM = hypertrophic cardiomyopathy; LVOT = left ventricular outflow tract obstruction; BP = blood pressure; HR = heart rate; CCBs = calcium channel blockers.

Drug Class	Examples	Role in HCM	Role in Hypertension	Key Considerations
Beta-blockers (non-vasodilating)	Metoprolol, Bisoprolol, Nadolol	First-line for symptomatic obstructive HCM, reduce LVOT gradient, anti-arrhythmic	First-line antihypertensive, controls HR and BP	Avoid in severe conduction abnormalities; beneficial dual use
Non-dihydropyridine CCBs	Verapamil, Diltiazem	Alternative for symptom relief in HCM, improve diastolic function	Used if intolerance to beta-blockers	Use cautiously with hypotension or severe LVOT obstruction
Dihydropyridine CCBs	Nifedipine, Amlodipine	Generally avoided in HCM due to vasodilation worsening LVOT obstruction	Common first-line for hypertension	Avoid in obstructive HCM; useful in non-obstructive hypertension
Diuretics (Loop, Thiazide)	Furosemide, Hydrochlorothiazide	Used cautiously for congestion relief	Central in hypertension management	Careful titration to avoid hypovolemia and LVOT worsening
Cardiac myosin inhibitors	Mavacamten, Aficamten	Target underlying HCM pathophysiology, reduce LVOT gradient	No direct antihypertensive effect but enables use of afterload reducers	Emerging therapy; need longer-term safety and efficacy data
SGLT2 inhibitors	Empagliflozin	Investigational use in HCM for improving metabolism and remodeling	Mild antihypertensive effect, cardioprotective	Promising in HCM; benefits still under study
Angiotensin receptor blockers (ARBs)	Valsartan	Limited evidence; potential to slow remodeling in early HCM	Key antihypertensive class	Not first-line for HCM, may be considered in selected younger patients

## 6. Discussion

In recent years, a remarkable shift in the epidemiology of hypertrophic cardiomyopathy (HCM) has been observed. The number of genotype-positive (G+) patients, typically characterized by earlier onset and higher arrhythmic risk, has remained relatively stable, while the expansion of cohort size is mainly driven by the inclusion of older, genotype-negative (G-) patients with sporadic disease, milder phenotype and an increased burden of cardiovascular comorbidities. These distinct clinical patterns generate the hypothesis that G- HCM may represent a distinct disease entity, mediated to a significant extent by acquired risk factors rather than solely by inherited genetic variants.

Arterial hypertension has emerged as the most frequent coexisting condition, with important implications for the diagnosis, prognosis, and therapeutic management of HCM. It has been associated with increased penetrance in gene variant carriers with one standard deviation increase in diastolic blood pressure, conferring a four-fold risk of HCM in genotype-negative individuals [64]. Notably, both the Mayo HCM Genotype Predictor and the Toronto Genotype Score identify a history of arterial hypertension as a negative predictor of genotype-positive status when estimating the likelihood of a pathogenic variant [65,66]. At the same time, hypertension may serve as a diagnostic confounder, potentially delaying the recognition of hypertrophic cardiomyopathy, while its presence also bears prognostic significance, being associated with poorer functional status.

The co-management of HCM and hypertension poses a considerable clinical challenge [6] and necessitates a nuanced, patient-centered strategy that addresses both conditions while minimizing detrimental hemodynamic consequences. Current clinical guidelines advocate for blood pressure targets consistent with general primary prevention standards, aiming for values below 130/80 mmHg to mitigate cardiovascular risk without worsening LVOT obstruction or diastolic dysfunction. In hypertensive patients exhibiting the classic obstructive HCM phenotype, β-blockers are recommended as first-line agents due to their dual role in decreasing myocardial contractility and heart rate, thereby reducing LVOT gradients and effectively controlling BP [67]. When β-blockers are contraindicated or poorly tolerated, non-dihydropyridine calcium channel blockers, including verapamil and diltiazem, serve as viable alternatives by enhancing diastolic relaxation and lowering resting LVOT gradients [9]. Antihypertensive therapy in patients with LVOT gradients should be cautiously adjusted, typically limiting or avoiding vasodilators like ACE inhibitors or ARBs. In non-obstructive phenotypes with preserved blood pressure, hypertension can be managed with standard antihypertensives, including ACE inhibitors, ARBs and diuretics, especially when diastolic dysfunction is advanced and blood pressure control is paramount. In patients with combined cardiometabolic burdens, adding agents such as SGLT2 inhibitors and GLP-1 receptor agonists may afford additional metabolic and cardiovascular benefits alongside blood pressure management. Patients presenting with advanced heart failure manifestations require prioritization of symptom management and carefully calibrated antihypertensive adjustments to avoid hypotension [68]. Achieving BP targets often necessitates combination antihypertensive therapies, underscoring the critical role of regular clinical monitoring and individualized therapy adjustments. Ultimately, a personalized approach that considers each patient’s phenotypic expression, symptom burden, and comorbidities is essential to optimizing clinical outcomes in patients with coexisting HCM and hypertension.

## 7. Conclusions

Hypertension and HCM frequently coexist, with prevalence estimates suggesting that nearly half of patients with HCM also suffer from arterial hypertension. Despite this frequent overlap, the interaction between these two conditions remains insufficiently explored. Data regarding potential mechanistic links, long-term outcomes, and optimal pharmacologic strategies remain limited, highlighting an important field for future research aimed at refining therapeutic approaches and improving patient outcomes.

## Data Availability

The data supporting the findings of this study are available from the sources cited in the references.
